# Methodological considerations for estimating indirect costs in children and adolescents with chronic conditions: a scoping review

**DOI:** 10.1186/s12887-024-05384-9

**Published:** 2025-01-29

**Authors:** Anne Kitschen, Lulseged M. Asegu, Dirk Sauerland

**Affiliations:** Chair for Institutional Economics and Health Policy, Department of Philosophy, Politics and Economics, Witten/Herdecke University, Witten, Germany

**Keywords:** Indirect costs, Children, Chronic diseases, Economic evaluations

## Abstract

**Background:**

In children and adolescents, the prevalence of chronic diseases, e.g., obesity, asthma, and attention-deficit/hyperactivity disorder (ADHD), has increased in the last decades. These diseases have negative effects on patients and their families and pose a significant economic burden. Indirect costs related to caregivers’ lost workdays or children’s and adolescents’ missed education are likely to be high. However, there are no guidelines for measuring and valuing indirect costs in this population. Thus, this scoping review aims to examine methods in published articles, compare these approaches, and analyze benefits and shortcomings.

**Methods:**

The systematic literature search was conducted in Medline, PsycINFO, Embase, NHS EED, and the HTA Database considering all articles from inception until 16 October 2024. Two researchers independently screened title, abstract, and full text. Cost-of-illness studies (COIs) reporting indirect costs for obesity, asthma, or attention-deficit/hyperactivity disorders (ADHD) in children and adolescents up to 24 years were included. Only studies published in English or German were considered. Methodological characteristics, measurement of indirect costs, cost components, data source, and costing methods were extracted.

**Results:**

The literature search revealed 45 studies. Thirty-two articles on asthma, eight on ADHD, and five on obesity were included. While all studies included absenteeism, only a few assessed and valued unpaid work (31.1%) or presenteeism (13.3%). Overall, 88.9% of the studies considered indirect costs for caregivers. Additionally, 51.1% considered productivity losses for children and adolescents, with 47.8% of these studies assigning a monetary value to these losses. The largest share of studies (53.3%) considered indirect costs by measuring and valuing caregivers’ lost work time.

**Discussion:**

In conclusion, various methodologies were used to consider indirect costs for chronic diseases in children and adolescents, underlining the need for standardization. This scoping review presents methodologies for incorporating indirect costs in COIs and other types of economic evaluations, which focus on children and adolescents and adopt a societal perspective. These indirect costs include both paid and unpaid activities, as well as absenteeism and presenteeism, not only for caregivers but also for children and adolescents themselves.

**Supplementary Information:**

The online version contains supplementary material available at 10.1186/s12887-024-05384-9.

## Background

In recent decades, the epidemiological profile of pediatric diseases has undergone a significant transformation. The incidence of communicable diseases among infants, children, and adolescents has declined, giving way to a rise in non-communicable diseases [1]. In particular, obesity, asthma, and attention-deficit/hyperactivity disorders (ADHD) have emerged as prevalent chronic conditions within this population. A meta-analysis estimated a global prevalence of 8.5% for obesity, 14.8% for overweight, and 22.2% for excess weight in children and adolescents [2]. Moreover, a higher prevalence of obesity in this population was found in high-income countries and regions [2]. The global prevalence of ADHD in children and adolescents was estimated to be 7.4% in a further meta-analysis [3]. The prevalence of asthma is estimated to be 8.1% among children and adolescents younger than 18 years in the United States in 2006–2018 [4]. The global prevalence for severe asthma in children and adolescents was estimated to be 3% [5]. Chronic conditions can have several negative effects on patients and their families, such as a lower health-related quality of life [6, 7], family dysfunctioning [7], emotional and behavioral problems [7, 8], and poorer school experiences [9] as well as a lower academic attainment [10]. In addition to these negative effects on children and adolescents and their families, chronic conditions are associated with a substantial economic burden to society [11–13]. Indirect costs, i.e., productivity losses often account for a large share of the overall costs in children and adolescents with chronic diseases [14–16]. The factors contributing to indirect costs vary by disease: ADHD is linked to a poor school performance [17], asthma to frequent absences from school [18], and obesity primarily associated with long-term productivity losses [13].

There are several cost-of-illness studies (COIs) analyzing the costs associated with chronic conditions in children and adolescents [14, 15, 19]. These studies aim to inform decision makers and thus support decisions on resource allocation [20]. When conducting COIs, various perspectives can be adopted and each perspective is accompanied with a specific range of costs included in the analysis [21]. The most common perspectives are the healthcare payer and the broader societal perspective [22]. Typically, when adopting the healthcare payer’s perspective, all costs paid or reimbursed by healthcare payers are relevant [21]. These primarily include direct medical costs, such as physician visits, hospital stays, and medications, which are reimbursed by the payer [21]. From a societal perspective, the analysis includes all healthcare-related expenses, regardless of the payer [21]. Accordingly, this perspective includes direct medical costs, direct non-medical costs as well as indirect costs, such as productivity losses [21]. Costs related to productivity loss include those for paid and unpaid production loss due to illness, disability, or premature death [23]. Thus, when interpreting results, it is important to consider the perspective adopted and the associated costs analyzed. Although the societal perspective is the most comprehensive one offering numerous reasons for adoption [24], not all included costs may be relevant from other perspectives, e.g., the healthcare payer's perspective [21].

There are two common approaches for estimating the costs of lost productivity. First, the human capital method (HCM) and second, the friction cost method (FCM) [25]. With regard to the HCM, productivity losses are typically calculated by using gross earnings. This approach is based on the assumption that the indirect costs of a disease are just as high as the loss of labor potential, which results in an economy through absenteeism (absence from work) or presenteeism (reduced productivity at work) [26]. When applying the FCM, the productivity loss is estimated based on the time span that an employer needs to fill the vacant position, which resulted from the respective disease [25]. The application of these approaches in the context of chronic conditions in children and adolescents allows for the measurement and valuation of productivity losses for caregivers, who may be unable to pursue work or other activities due to their child’s disease. The assessment and valuation of indirect costs for children and adolescents themselves, such as school absences, is more challenging. Nevertheless, it is crucial to acknowledge that a lack of academic attainment may result from absences from school caused by chronic conditions [10]. Such absences may lead to productivity losses in the form of future earning losses [27]. Although indirect costs often account for a large share of the overall costs in children and adolescents with chronic diseases [11, 14–16], these are frequently underrepresented in economic analyses focusing on this population [11, 14, 15]. Furthermore, research on appropriate methodologies or guidelines to value indirect costs in children and adolescents and their caregivers is limited. Although there are several systematic reviews analyzing the economic burden of chronic conditions [14, 15, 19], there is no systematic review focusing on the methodologies to assess and value indirect costs in this population. Consequently, this scoping review attempts to provide an overview of the methodologies applied in existing publications to estimate the indirect burden for children and adolescents, as well as for caregivers, considering one of three common chronic childhood diseases, i.e., asthma, ADHD, and obesity.

## Methods

### Literature search and selection criteria

This scoping review follows the Preferred Reporting Items for Systematic Reviews and Meta-Analyses Extension for Scoping Reviews (PRISMA-ScR) [28].

The literature search was conducted in Medline, PsycINFO, Embase, NHS EED, and the HTA Database considering all articles published from inception to 16 October, 2024. The search strategy consisted of three parts: the first was to identify studies including children and adolescents: child* OR pediatric* OR adolescen* OR infant* OR pediatric* OR juvenile* OR young person* OR young adult* and the second was to identify studies analyzing indirect costs: ((indirect OR productivity OR societal OR economic) AND (cost* OR loss* OR burden)) and the third part was to detect the aforementioned relevant diseases: (asthma OR obesity OR overweight OR adhd OR attention deficit hyperactivity disorder*). In order to detect all relevant articles, following exploded MeSH-Terms were used: *child, adolescent, infant, asthma,* and *obesity*. As the initial three were not available for the search in PsycINFO these were substituted with *child health* and *adolescent health* for the search in this database. The comprehensive search strategy is presented in the supplementary material.

**Fig. 1:**
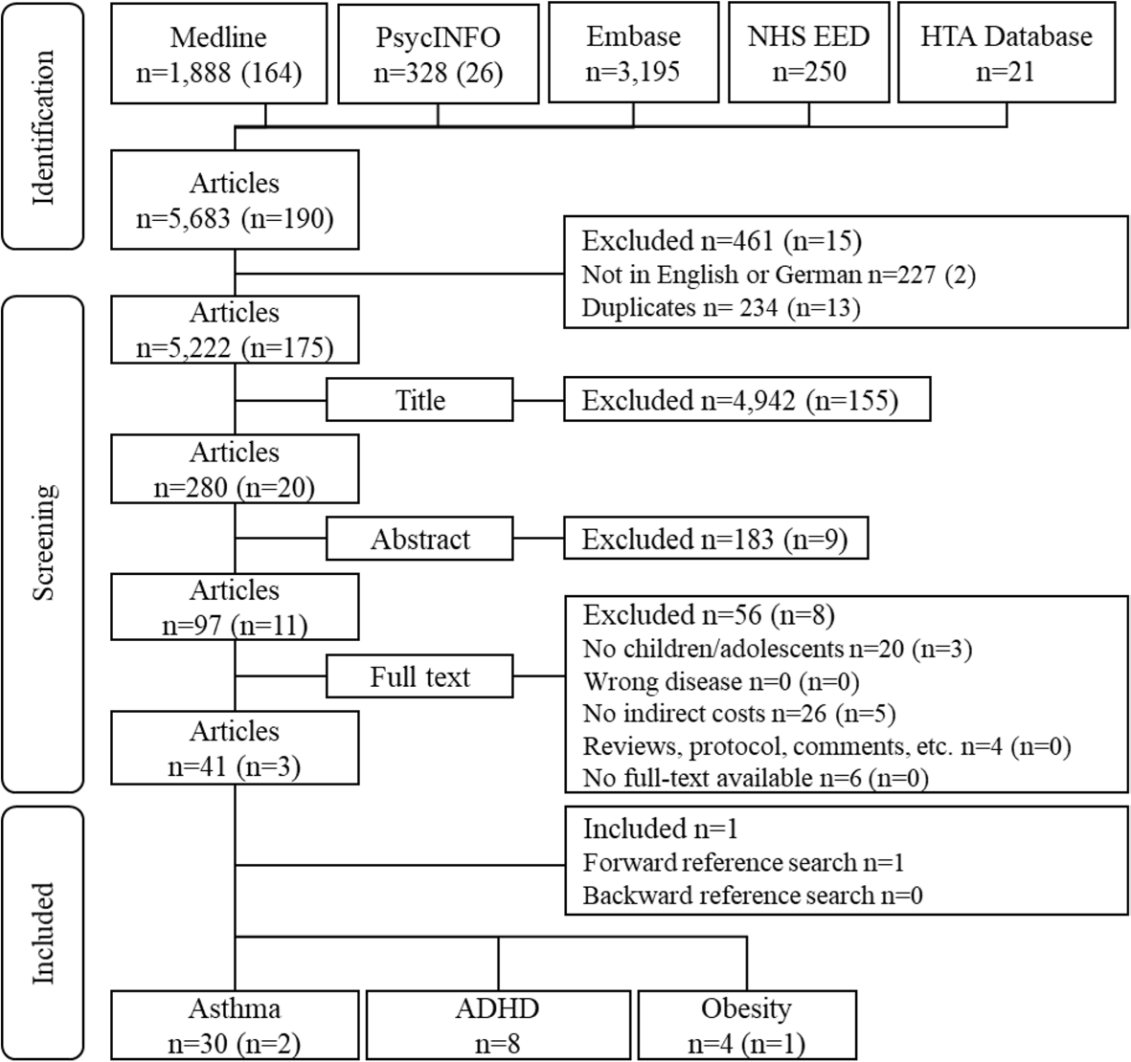
Flowchart

COIs examining children and adolescents up to 24 years suffering from asthma, ADHD, or obesity and valuing indirect cost monetarily were included in the analysis. The age limit was chosen according to a recent definition of adolescence [29]. The specific diseases were chosen as these are the most common chronic conditions among children and adolescents [4, 30, 31]. Furthermore, they are associated with various types of economic burden, including a poor school performance [17], frequent school absences [18], and long-term productivity losses [13]. Thus, by including these prevalent diseases, we aimed to capture diverse causes of indirect cost and differing methodologies to assess and value these. Specific study types, such as systematic reviews, protocols, editorials, comments, and letters were excluded. Grey literature was excluded to focus on peer-reviewed methods utilized in academic research, ensuring the reliability and validity of the data analyzed. Only studies published in German or English were considered. Two researchers (AK, LA) independently screened title, abstract, and full text. Any differing decisions were discussed. If no consensus was reached by discussion, a third researcher was involved (DS). In addition, a forward and backward reference search was conducted to identify further relevant articles. The screening process is displayed in a PRISMA flow diagram (Fig. 1).

Methodological study characteristics, i.e., country of analysis, analyzed disease, included types and measures of indirect costs, data sources, perspective, and costing method, were extracted. All data were extracted into a structured form by two independent researchers (AK, LA). Differences in extracted data were adjusted by a repeated comparison to the data in the original article. As this is a scoping review providing an overview of different methodologies and not the results, we did not assess the quality of the included studies [32].

## Results

### Search results

The literature search revealed 5,873 results published up to October 16, 2024 (Fig. 1). Of these, 247 were duplicates and 229 were not published in English or German. The reasons for excluding studies during the full-text screening were that 23 studies did not focus on children and adolescents, and 31 studies did not report indirect costs. Furthermore, no full-text was available for further six studies. After eligibility assessment, 44 articles were included for the in-depth analysis from the database search. In addition, one further article was found in the forward reference search. Finally, 32 articles on asthma [33–63], 8 on ADHD [64–71], and 5 on obesity [13, 72–75] met the inclusion criteria and were included in this scoping review.

### Methodological study characteristics

Twenty of the included studies were from the United States [33, 34, 36, 43, 46, 47, 49, 52, 53, 55, 57, 59, 62–64, 69–71, 74, 76], four from Germany [13, 56, 66, 72], three from the United Kingdom [39, 58, 75], two each from Canada [40, 61], Turkey [44, 45], and one each from France [54], Switzerland [60], Spain [68], Finland [50], Sweden [67], Netherlands [65], Singapore [51], Portugal [48], Iran [42], Korea [41], Hungary [38], Australia [73], and Nigeria [35] (Table 1). A further included European study [37] estimated costs for six different countries, i.e., Austria, Belgium, Italy, Slovenia, Spain, and Sweden. All but one study [65] used the HCM to estimate indirect costs. Hakkaart et al. [65] calculated indirect costs based on the FCM. Two studies [33, 34] did not report any details on the costing method. All studies included absenteeism and further six studies [13, 38, 42, 65, 69, 71] (13.3%) also considered presenteeism. Unpaid work was considered for indirect costs in 14 of the included studies (31.1%) [13, 36, 39–41, 46, 47, 57, 58, 63, 68, 69, 71, 73]. A further study included unpaid [65] work but did not value it monetarily. The included studies used data from different sources for estimating the quantity of productivity loss, e.g., school days or workdays lost. Most studies derived their data on productivity loss quantity from survey data, such as the Medical Expenditure Panel Survey (MEPS) [33, 36, 46, 47, 52, 53, 57, 62, 64, 74, 76], the National Health Interview Study (NHIS) [63], or the Canadian Health Survey [40]. Other studies draw their data from administrative data sources, i.e., data from the employer data [34, 70], the Office for National Statistics [75], medical/case reports [39, 43, 45], or the national health insurance database [41]. Further data sources were questionnaires in 14 studies [35, 42, 44, 48, 51, 54, 55, 59, 65, 67, 68, 71–73], secondary literature in seven studies [13, 43, 46, 47, 62, 69, 75], interviews in five studies [38, 45, 49, 58, 61], and a parent-held diary in a further study [39]. Five studies [42, 51, 59, 65, 71] using questionnaires gave further information on these. The questionnaires were the Caregiver Indirect and Informal Care Cost Assessment Questionnaire [77], the TiC-P Questionnaire [78], the Work Productivity and Activity Impairment instrument [79, 80], and The Impact Questionnaire [71]. To value the quantity of productivity in monetary terms, most of the studies [34, 38–41, 43, 46–48, 50, 53–58, 60, 64, 66–74] used data on average wage provided by the government or the employer. Other studies drew their data for valuation from survey data [33, 36, 52], secondary literature [13, 49, 58, 59, 62, 63], questionnaires [51], or the International Monetary Fund [42].

**Table 1:** Methodological characteristics

Study	Country	Disease	Costing method	Cost component	Data source for quantity	Data source for valuation
Barnett et al. 2010 [36]	USA	Asthma	HCM	Absenteeism, unpaid work	MEPS	MEPS (Caregiver’s wage, wage for household work)
Belova et al. 2020 [33]	USA	Asthma	NR	Absenteeism	MEPS; model-based calculated	MEPS (Caregiver's average wage)
Beyhun et al. 2007 [44]	Turkey	Asthma	N/A	Absenteeism	Questionnaire	N/A
Beyhun et al. 2009 [45]	Turkey	Asthma	N/A	Absenteeism	Face-to-face interview based on a structured questionnaire; chart review	N/A
Brandt et al. 2014 [46]	USA	Asthma	HCM	Absenteeism, unpaid work	MEPS; secondary literature	US Census Bureau (average wage)
Brandt et al. 2012 [47]	USA	Asthma	HCM	Absenteeism, unpaid work	MEPS; secondary literature	US Census Bureau (average wage)
Breitfelder et al. 2011 [72]	Germany	Obesity	HCM	Absenteeism	Questionnaire	Federal statistical office (age and sex-specific average wage)
Carrello et al. 2021 [73]	Australia	Obesity	HCM	Absenteeism, unpaid work	Questionnaire	Bureau of Statistics (average wage)
Chanel et al. 2016 [37]	Europe^a^	Asthma	HCM	Absenteeism	NR	NR
Ferreira de Magalhaes et al. 2017 [48]	Portugal	Asthma	HCM	Absenteeism	Questionnaire	Ministry of Social Security (profession-specific monthly income)
Finkelstein et al. 2021 [51]	Singapore	Asthma	HCM	Absenteeism	Caregiver Indirect and Informal Care Cost Assessment Questionnaire [12]	Caregiver Indirect and Informal Care Cost Assessment Questionnaire [12] (Caregiver's average wage)
Gendo al. 2003 [49]	USA	Asthma	HCM	Absenteeism	Interview	Secondary literature
Gupte-Singh et al. 2017 [64]	USA	ADHD	HCM	Absenteeism	MEPS	Bureau of Labor Statistics (average wage)
Hakkaart van Roijen et al. 2007 [65]	Netherlands	ADHD	FCM	Absenteeism, presenteeism, unpaid work (not valued)	Trimbos and iMTA questionnaire on Costs associated with Psychiatric illness [16]	NR (average wage)
Herjavecz et al. 2003 [38]	Hungary	Asthma	HCM	Absenteeism, presenteeism	Interview	Hungarian Office of Statistics (average wage)
Kennedy et al. 2023 [39]	UK	Asthma	HCM	Absenteeism, unpaid work	Case report form; parent-held diaries	Office of National Statistics (age-specific average wage, average wage for housework, volunteering and informal care); Leisure time: Department of Transport (value of non-working time)
Kleinman et al. 2009 [81]	USA	Asthma	NR	Absenteeism	Administrative data from employer	Administrative data from employer
Korhonen et al. 2001 [50]	Finland	Asthma	HCM	Absenteeism	Assumption: Length of hospital stay and three days care after discharge	Finnish Office of Statistics (average wage)
Kotsopoulos et al. 2013 [66]	Germany	ADHD	HCM	Absenteeism	Administrative data	Administrative data
Krahn et al. 1996 [40]	Canada	Asthma	HCM	Absenteeism, unpaid work	Canadian Health Survey	Paid work: Survey of Employment, Payrolls, and Hours (average wage); Unpaid work: estimations based on secondary data sources (value of housekeeping services)
Lee et al. 2011 [41]	Korea	Asthma	HCM	Absenteesim, unpaid work	National health insurance claims database	Ministry of Labor (age and gender specific average wage)
Lightwood et al. 2009 [74]	USA	Obesity	HCM	Absenteeism	MEPS	United States Census Bureau (age and gender specific average wage)
Nurmagambetov et al. 2017 [53]	USA	Asthma	HCM	Absenteeism	MEPS	Bureau of Labor Statistics (state, age, and sex-specific average wage)
Nurmagambetov et al. 2018 [52]	USA	Asthma	HCM	Absenteeism	MEPS	MEPS (Caregivers’ average wage)
Nyden et al. 2008 [67]	Sweden	ADHD	HCM	Absenteeism	Questionnaire	Swedish social insurance agency (average wage)
Ochoa-Moreno et al. 2024 [75]	UK	Obesity	HCM	Absenteeism	Office for National Statistics; Secondary data source [28] for mortality rates	Office of National Statistics (average wage)
Pamuk et al. 2021 [12]	France	Asthma	HCM	Absenteeism	Questionnaire	National Institute of Statistics and Economic Studies in France (average wage)
Quintero et al. 2018 [68]	Spain	ADHD	HCM	Absenteeism, unpaid work	Questionnaire	Spanish National Institute of Statistics (average wage)
Schein et al. 2022 [69]	USA	ADHD	HCM	Absenteeism, presenteeism,unpaid work	Secondary literature	United States Census Bureau (average wage)
Schmier et al. 2007 [55]	USA	Asthma	HCM	Absenteeism	Questionnaire	Bureau of Labor Statistics
Schramm et al. 2003 [56]	Germany	Asthma	HCM	Absenteeism	Interview	Statistical Yearbook (average wage)
Seyedrezazadeh et al. 2023 [42]	Iran	Asthma	HCM	Absenteeism, presenteeism	Work Productivity and Activity Impairment instrument [35]	International Monetary Fund
Smith et al. 1997 [57]	USA	Asthma	HCM	Absenteeism, unpaid work	MEPS	Paid work: Bureau of Labor (average wage Unpaid work: Bureau of Labor (average wage for private household services)
Song et al. 2021 [76]	USA	Asthma	HCM	Absenteeism	MEPS	MEPS (average wage)
Sonntag et al. 2015 [13]	Germany	Obesity	HCM	Absenteeism, presenteeism, unpaid work	Secondary literature; Model-based (markov)	Secondary literature
Stevens et al. 2003 [58]	UK	Asthma	HCM	Absenteeism, unpaid work	Structured interview	Paid work: Office for National Statistics Paid work (mean sex-specific wages); Unpaid work: Secondary literature (mean wage for home-care worker)
Swensen et al. 2003 [70]	USA	ADHD	HCM	Absenteeism	Administrative data from employer (Caregivers ‘ wage)	Administrative data from employer (Caregivers ‘ wage)
Szefler et al. 2011 [59]	USA	Asthma	HCM	Absenteeism	Work Productivity and Activity Impairment instrument [42]	Secondary literature (sex-specific average wage)
Szucs et al. 1999 [60]	Switzerland	Asthma	HCM	Absenteeism	NR	Swiss Office of Statistics (average wages)
Ughasoro et al. 2021 [35]	Nigeria	Asthma	HCM	Absenteeism	Questionnaire	NR
Ungar et al. 2001 [61]	Canada	Asthma	HCM	Absenteeism	Interview	Caregivers’ wage
Wang et al. 2005 [62]	USA	Asthma	HCM	Absenteeism	MEPS; secondary literature	Secondary literature (average wage)
Weiss et al. 1992 [63]	USA	Asthma	HCM	Absenteeism, unpaid work	NHIS	Paid work: caregivers’ annual wage; Unpaid work: secondary literature (average wage for housework)
Zarate-Gonzalez et al. 2024 [43]	USA	Asthma	HCM	Absenteeism	Patient discharge data; secondary data source [49]	California Employment Development Department; Bureau of Labor Statistics
Zhao et al. 2019 [71]	USA	ADHD	HCM	Absenteeism, presenteeism, unpaid work	The Impact Questionnaire [50]	Bureau of Labor Statistics (average wage)

*ADHD* Attention-Deficit/Hyperactivity Disorder, *HCM* Human Capital Method, *FCM* Friction Cost Method, *MEPS* Medical Expenditure Panel Survey, *NHIS* National Health Interview Study

NR = not reported; N/A = not available

^a^estimated for following countries: Austria, Belgium, Italy, Slovenia, Spain, and Sweden

### Measurement of indirect costs

The methodology applied to estimate indirect costs varied across the included studies (Table 2). The majority of the studies based their estimation of indirect costs on two measures [36, 42–45, 53–56, 59, 61–63, 65, 67, 68, 70, 73, 81] (42.2%) or on one measure [35, 37, 40, 41, 48–51, 58, 60, 64, 66, 72, 74–76] (35.6%). Ten studies [13, 33, 38, 39, 46, 47, 52, 57, 69, 71] (22.2%) included three different measures. All but five studies [13, 42, 66, 74, 75] considered the productivity losses for caregivers and 23 studies (51.1%) [13, 36, 38, 39, 41–47, 52–55, 59, 62, 63, 66, 69, 73, 75] for children and adolescents themselves whereas 11 of these studies [13, 39, 41–43, 52, 62, 63, 66, 69, 75] valued the losses in monetary terms.

**Table 2:** Measurement of indirect costs

	Lost work time	Lost school days (proxy for caregiver's lost work day)	Time spent for care	Long-term inability to work	Reduced efficiency at work	Impact of disease on earnings	Impact of disease on occupational choice	Lost school time^a^	Lifetime earnings	Lost work/school time	Reduced efficiency at work/school
	**Caregivers**							**Children/Adolescents**			
Barnett et al. 2010 [36]		●						●			
Belova et al. 2020 [33]				●^d^		●^d^	●^a,d^				
Beyhun et al. 2007 [44]	●^a^							●			
Beyhun et al. 2009 [45]	●^a^							●			
Brandt et al. 2014 [46]		●	●					●			
Brandt et al. 2012 [47]		●	●					●			
Breitfelder et al. 2011 [72]	●										
Carrello et al. 2021 [73]		●						●			
Chanel et al. 2016 [37]	●										
Ferreira de Magalhaes et al. 2017 [48]	●										
Finkelstein et al. 2021 [51]	●										
Gendo al. 2003 [49]	●										
Gupte-Singh et al. 2017 [64]		●									
Hakkaart van Roijen et al. 2007 [65]	●				●						
Herjavecz et al. 2003 [38]	●							●			●^a^
Kennedy et al. 2023 [39]	●		●							●	
Kleinman et al. 2009 [81]	●			●							
Korhonen et al. 2001 [50]	●										
Kotsopoulos et al. 2013 [66]									●^c^		
Krahn et al. 1996 [40]		●									
Lee et al. 2016 [41]			●								
Lightwood et al. 2009 [74]									●^d^		
Nurmagambetov et al. 2018 [52]		●						●	● ^d^		
Nurmagambetov et al. 2017 [53]		●						●			
Nyden et al. 2008 [67]	●		●^a^								
Ochoa-Moreno et al. 2024 [75]									● ^d^		
Pamuk et al. 2021 [12]	●							●			
Quintero et al. 2018 [68]	●		●								
Schein et al. 2022 [69]			●						●^e^	●^e^	
Schmier et al. 2007 [55]	●							●^c^			
Schramm et al. 2003 [56]	●			●							
Seyedrezazadeh et al. 2023 [42]										●	●
Smith et al. 1997 [57]	●	●	●								
Song et al. 2021 [76]		●									
Sonntag et al. 2015 [13]									●^d^	●^d^	●^d^
Stevens et al. 2003 [58]			●^b^								
Swensen et al. 2003 [70]	●			●							
Szefler et al. 2011 [59]		●						●			
Szucs et al. 1999 [60]	●										
Ughasoro et al. 2021 [35]	●										
Ungar et al. 2001 [61]	●		●								
Wang et al. 2005 [62]	●								●		
Weiss et al. 1992 [63]		●							●		
Zarate-Gonzalez et al. 2024 [43]			●							●	
Zhao et al. 2019 [71]	●				●^a^	●					
% of studies	53.3	26.7	24.4	8.9	4.4	4.4	2.2	26.7	11.1	6.7	17.8

^a^not valued monetarily; ^b^used as proxy for lost waged and unwaged activities; ^c^measured categorically; ^d^forecast employing a model; ^e^only for working patients

The majority of studies [34, 35, 37–39, 44, 45, 48–51, 54–57, 60–62, 65, 67, 68, 70–72] (53.3%) assessed caregivers’ work loss due to the child's illness to calculate indirect costs. Twelve studies [36, 40, 46, 47, 52, 53, 57, 59, 63, 64, 73, 76] (26.7%) assessed the school days lost as an indicator for the number of workdays lost by the caregivers. Eleven studies [39, 41, 43, 46, 47, 57, 58, 61, 67–69] (24.4%) considered the caregivers’ time spent for care of the child. Caregivers’ long-term inability to work due to the child’s disease was analyzed and valued monetarily in four studies [33, 34, 56, 70] (8.9%). In addition to that, two studies each (4.4%) included caregivers’ reduced efficiency at work [65, 71] and the impact of disease on caregivers’ earnings [33, 39, 67, 71]. One study [33] analyzed the impact on the caregivers’ occupational choice.

When considering productivity losses for children and adolescents themselves, most of the studies [36, 38, 44–47, 52–55, 59, 73] (26.7%) reported the mean number of school days lost without valuing these monetarily. Eight studies [13, 41, 52, 62, 63, 66, 69, 75] (17.8%) calculated children’s and adolescents’ loss of lifetime earnings due to premature mortality or morbidity.

## Discussion

The objective of this scoping review was to synthesize the existing methodological approaches to measure and value indirect costs in children and adolescents with chronic conditions. The review yielded three main findings: First, lost unpaid work was only considered in 14 studies [13, 36, 39–41, 46, 47, 57, 58, 63, 68, 69, 71, 73] (31.1%) and caregivers’ time spent for care were considered in only 11 studies [39, 41, 43, 46, 47, 57, 58, 61, 67–69] (24.4%). Second, the most common approach to estimate indirect costs across the studies was to survey and value the caregivers’ work time lost or children’s and adolescents’ lost school days as an indicator for lost work days and to multiply this by an average wage. Third, a total of 11 studies (24.4%) [13, 39, 41–43, 52, 62, 63, 66, 69, 75] considered indirect costs for children and adolescents themselves while also valuing these monetarily.

Similar results were found in another systematic literature review [82], analyzing and assessing measurement instruments for productivity losses and unpaid work while not focusing on children and adolescents. Hubens et al. [82] found that 76.2% of the instruments applied in the included studies contained questions on absenteeism, also 76.2% on presenteeism, and 33.3% on unpaid work. This disregard of unpaid work has already been discussed in a previous systematic review [23]. Krol et al. [23] recommend the consideration of unpaid work for specific diseases due to the given societal value of unpaid labor. The relevance of considering unpaid work in caregivers of children and adolescents with chronic diseases results from studies finding a significant impact of chronic diseases on the parental choice not to work or to work less [83]. Consequently, the standard approach applied in the majority of studies neglects the indirect costs caused by caregivers leaving formal labor force to informally care for children with chronic conditions. Moreover, the second most common approach was to value children’s and adolescents’ school days lost as an indicator for the caregivers’ lost work time due to the chronic condition. This approach does not take into account the care time that occurs outside of school hours, e.g., for physician visits after school. During this time, caregivers may have productivity losses in paid or unpaid work regardless of whether the child attended in school or not. Nevertheless, five of the included studies [46, 47, 58, 68, 69] estimated indirect costs based on caregivers’ time spent for care while considering paid as well as unpaid work.

A total of 11 studies [13, 39, 41–43, 52, 62, 63, 66, 69, 75] estimated indirect costs for children and adolescents themselves while also valuing these monetarily. For future COIs, various approaches can be considered to account for indirect costs in children and adolescents themselves [84]. Andronis et al. [84] summarized two types of time forgone, which could be considered in COIs including children and adolescents who are not yet part of the working population: First, childhood education, and second, lost leisure time.

Leisure time includes “all activities that we cannot pay somebody else to do for us and that we do not really have to do at all if we do not wish to.” [85] Losses in leisure time can be valued assuming that the marginal income from working for an additional hour is also the marginal value of an additional hour of leisure, the opportunity cost of forgone leisure time is equal to the prevailing hourly wage rate [86]. However, in cases employed work is not an alternative in children and adolescents a justifying argument for this approach is missing. As further option to value leisure time in children and adolescents, Andronis et al. [84] suggest to ask individuals how much they are willing to pay for an additional hour of leisure. Hereby it is questionable if children have the cognitive capacity or experience to understand and answer this question regarding the willingness to pay for leisure time [87]. To address this issue, a feasible solution would be to interview caregivers or parents about how much they value an additional hour of their children's leisure time. While these assessments can offer useful insights, they may not fully reflect the true value that leisure time holds for children and adolescents themselves.

A further approach could be the consideration of losses in formal education as chronic health conditions are associated with a lower academic attainment [10], which may also result in lower future earnings [27]. To value education losses based on the lower educational attainment and losses in future earnings is consistent with the HCM [84]. There are two publications that calculated and reported a potential basis for this approach of valuation. Cattan et al. [88] estimated in a Swedish study that the short-term effect of an additional ten days of school absence on school performance can be translated into a decrease in yearly earning potential of 43.25 USD based on values of 2002. Hanushek and Woessmann [27] calculated pooled individual income losses for OECD countries, which ranged between 1.9% and 7.7% for 0.25 and 1 lost school-year equivalent, respectively.

### Strengths and limitations

The existing literature on methodological approaches in children and adolescents is limited. To the best of our knowledge, this is the first review focusing on methodological approaches of measuring and valuing indirect costs in children and adolescents. In addition, we identified the advantages and disadvantages of specific methodological approaches for estimating indirect costs of chronic diseases in children and adolescents and provided concepts for the inclusion of indirect costs in children and adolescents in future COIs. This way the current scoping review may encourage future studies to more consistently include indirect costs. Another strength of this work is that it was conducted in accordance with the PRISMA guideline, which enhances the transparency and supports the interpretation of the results. However, there are some limitations that must be considered when interpreting the results. We cannot draw a conclusion about the consequences of including or neglecting specific kinds of indirect costs as we focused on the methodological characteristics and did not extract and analyze the estimated amount of indirect costs. In addition, our study focused on COIs and on three specific common pediatric diseases. Studies focusing on other diseases and conducting other types of economic evaluations may provide further approaches for the inclusion of indirect costs. Nonetheless, systematic reviews focusing on either economic evaluations of interventions or on costs of chronic pain [14], ADHD [11, 89], or overweight [15] in children and adolescents generally confirm the inconsistency in inclusion of indirect costs. Other COIs focusing on economic burden of epilepsy [90] or diabetes [91], employed methodologies also used in the studies included in this review. Riechmann et al. [90] accounted for indirect costs associated with parental work absences, as well as decisions to quit or reduce working hours. Butt et al. [91] focused on the economic impact of absenteeism and premature mortality.

## Conclusion

This scoping review provides a comprehensive synthesis of methodological approaches for measuring and valuing indirect costs of chronic diseases in children and adolescents. Our findings highlight the great heterogeneity and complexity of estimating indirect costs underscoring the need for more standardized approaches in future COIs. Therefore, this review presents methods to include indirect costs, thereby enhancing the comprehensiveness of cost analyses in pediatric populations. The consistent inclusion of indirect costs is crucial, as they often account for a large share of total costs in children and adolescents with chronic diseases [11, 14–16]. However, only a limited number of studies in this field account for indirect costs [11, 14, 15]. Ignoring relevant costs might lead to an inefficient allocation of resources [24]. Therefore, future COIs or other types of economic evaluations adopting a societal perspective should comprehensively account for indirect costs. These include not only lost paid and unpaid work, as well as absenteeism and presenteeism in caregivers, but also the economic consequences of school absences and reduced productivity in children and adolescents.

## Supplementary Information

Supplementary Material 1.

## Data Availability

Not applicable. No datasets were generated or analyzed during the current study. All data that were extracted from the included studies are presented in the figures and tables.

## Abbreviations

ADHD: Attention-deficit/hyperactivity disorder
COI: Cost-of-illness study
HCM: Human Capital Method
FCM: Friction Cost Method
