# Supplementary material for: Methodological considerations for estimating indirect costs in children and adolescents with chronic conditions: a scoping review
Source: BMC Pediatr. 2025 Jan 29;25:73. doi: 10.1186/s12887-024-05384-9 (PMC11776138; doi:10.1186/s12887-024-05384-9)
Supplement: Supplementary file 1 — Supplementary Material 1. [file 12887_2024_5384_MOESM1_ESM.docx]

**Supplementary table – Search strategy**

|  | Medline  (via Ovid .ab,ti.) | PsycINFO  (via Ovid .ab,ti) | Embase  (via Ovid .ab.ti) | NHS EED  (via CRD .af) | HTA Database (via CRD .af) |
| --- | --- | --- | --- | --- | --- |
| #1 | exp Child/ | exp Child Health/ | exp Child/ | exp Child/ | exp Child/ |
| #2 | exp Adolescent/ | exp Adolescent health/ | exp Adolescent/ | exp Adolescent/ | exp Adolescent/ |
| #3 | exp Infant/ |  | exp Infant/ | exp Infant/ | exp Infant/ |
| #4 | "child*" | "child*" | "child*" | "child*" | "child*" |
| #5 | "adolescen*" | "adolescen*" | "adolescen*" | "adolescen*" | "adolescen*" |
| #6 | "infant*" | "infant*" | "infant*" | "infant*" | "infant*" |
| #7 | "pediatric*" | "pediatric*" | "pediatric*" | "pediatric*" | "pediatric*" |
| #8 | "juvenile*" | "juvenile*" | " juvenile*" | "juvenile*" | "juvenile*" |
| #9 | "young person*" | "young person*" | "young person*" | "young person*" | "young person*" |
| #10 | "young adult*" | "young adult*" | "young adult*" | "young adult*" | "young adult*" |
| #11 | 1 - 10/OR | 1 - 10/OR | 1 - 10/OR | 1 - 10/OR | 1 - 10/OR |
| #12 | „indirect“ | „indirect“ | „indirect“ | „indirect“ | „indirect“ |
| #13 | „productivity*“ | „productivity*“ | „productivity*“ | „productivity*“ | „productivity*“ |
| #14 | „societal*“ | „societal*“ | „societal*“ | „societal*“ | „societal*“ |
| #15 | „economic*“ | „economic*“ | „economic*“ | „economic*“ | „economic*“ |
| #16 | 12 – 15/OR | 12 – 15/OR | 12 – 15/OR | 12 – 15/OR | 12 – 15/OR |
| #17 | „burden“ | „burden“ | „burden“ | „burden“ | „burden“ |
| #18 | „loss“ | „loss“ | „loss“ | „loss“ | „loss“ |
| #19 | „cost*“ | „cost*“ | „cost*“ | „cost*“ | „cost*“ |
| #20 | 17 – 19/OR | 17 – 19/OR | 17 – 19/OR | 17 – 19/OR | 17 – 19/OR |
| #21 | 16 AND 20 | 16 AND 20 | 16 AND 20 | 16 AND 20 | 16 AND 20 |
| #22 | „asthma“ | „asthma“ | „asthma“ | „asthma“ | „asthma“ |
| #23 | exp asthma/ | exp asthma/ | exp asthma/ | exp asthma/ | exp asthma/ |
| #24 | „obesity“ | „obesity“ | „obesity“ | „obesity“ | „obesity“ |
| #25 | exp obesity/ | exp obesity/ | exp obesity/ | exp obesity/ | exp obesity/ |
| #26 | „overweight*“ | „overweight*“ | „overweight*“ | „overweight*“ | „overweight*“ |
| #27 | „adhd“ | „adhd“ | „adhd“ | „adhd“ | „adhd“ |
| #28 | „attention deficit hyperactivity disorder“ | „attention deficit hyperactivity disorder“ | „attention deficit hyperactivity disorder“ | attention deficit hyperactivity disorder“ | attention deficit hyperactivity disorder“ |
| #39 | 22 – 28/OR | 22 – 28/OR | 22 – 28/OR | 22 – 28/OR | 22 – 28/OR |
| #30 | 11 AND 21 AND 29 | 11 AND 21 AND 29 | 11 AND 21 AND 29 | 11 AND 21 AND 29 | 11 AND 21 AND 29 |
| #32 | (limit 30 to  dt=20220822-20241016)^a^ | (limit 30 to  up=20220822-20241016)^a^ | NA^b^ | NA^b^ | NA^b^ |
| .ab = abstract; .ti = title; * = truncation; .af = all fields; exp = explode MeSH-Term;  ^a^ used for search upated on October 16, 2024  ^b^ no update needed as first search was conducted at October 16, 2024 | | | |  |  |
